# Supplementary material for: The role of non-additive gene action on gene expression variation in plant domestication
Source: EvoDevo. 2023 Feb 10;14:3. doi: 10.1186/s13227-022-00206-4 (PMC9912502; doi:10.1186/s13227-022-00206-4)
Supplement: Supplementary file 1 — Additional file 1: Figure S1. GO enrichment analysis showing biological functions altered in the studied genetic crosses. Dot plots show the enrichment, or presence of each GO for each mode of gene action (Additive, Partially-Dominant, Dominant, Transgressive) across the array of genetic crosses. Color of dots indicate the significance of the enrichment; the size depicts the number of annotated genes to each GO. Figure S2. A sampling-based analysis revealed a uniform ~60X sequencing coverage for all the libraries used in the study. The per-transcript sequencing coverage (Log2 normalized read counts) is shown as boxplots for the three genotypes of each species. The line within the boxes depicts the median, whereas the dots outside the box represent outlier values. Table S1. Summary of the per-species analysis of differential expression between parental and F1 hybrids. Table S2. Transcripts associated to domestication-related funcitons showing non-additive gene action. Table S3. Summary of the pair-wise differences between discrete categories of gene action of transcription factors and their target genes. Table S4. Details of the per-species RNA-seq libraries used in the study. [file 13227_2022_206_MOESM1_ESM.docx]

**Additional file**

**Figure S2**. A sampling-based analysis revealed a uniform ~60X sequencing coverage for all the libraries used in the study. The per-transcript sequencing coverage (Log2 normalized read counts) is shown as boxplots for the three genotypes of each species. The line within the boxes depicts the median, whereas the dots outside the box represent outlier values.

**Figure S1**. GO enrichment analysis showing biological functions altered in the studied genetic crosses. Dot plots show the enrichment, or presence of each GO for each mode of gene action (Additive, Partially-Dominant, Dominant, Transgressive) across the array of genetic crosses. Color of dots indicate the significance of the enrichment; the size depicts the number of annotated genes to each GO.

**Tables**

**Table S1**

| **Species** | **Genetic cross** | **Divergence type** | **Expressed transcripts (F0s ∪ F1)** | **Transcripts showing DE between F0s** | **Portion of the transcriptome showing DE** | **DE transcripts showing expression in F1s** |
| --- | --- | --- | --- | --- | --- | --- |
| *Panicum hallii* | hallii-filipes | Natural Selection | 25127 | 346 | 1.3% | 346 |
| *Zea mays* | W22-TIL3 | Domestication | 62022 | 22092 | 35.61% | 22092 |
| *Zea mays* | Oh43-TIL3 |  | 62022 | 22602 | 36.44% | 22602 |
| *Zea mays* | B73-TIL3 |  | 62022 | 22560 | 36.37% | 22560 |
| *Capsicum annuum* | Puya-Chiltepin |  | 16998 | 7569 | 44.52% | 7569 |
| *Helianthus annus* | annus-petiolaris |  | 71362 | 13770 | 19.29% | 13770 |
| *Oryza sativa* | indica-japonica | Inbreds | 31471 | 2132 | 6.7% | 2132 |
|  | japonica-indica |  |  |  |  | 2132 |
| *Arabidopsis thaliana* | Col-C24 |  | 48360 | 7160 | 14.8% | 7160 |
|  | C24-Col |  |  |  |  | 7155 |
| *Arabidopsis thaliana* | Col-Ler |  | 48360 | 6838 | 14.13% | 6837 |
|  | Ler-Col |  |  |  |  | 6838 |

**Table S2**

| **Biological function** | **Divergence type** | **Number of transcripts** | **Median \|*k*\|** | **p-val Wilcoxon μ \|k\| > 0.25** |
| --- | --- | --- | --- | --- |
| Cell cycle | Natural Selection | 1 | NA | NA |
|  | Domestication | 171 | 0.8 | 3.73e-113 |
|  | Inbreds | 834 | 0.76 | 1.20e-26 |
| Cell fate | Natural Selection | 0 | NA | NA |
|  | Domestication | 22 | 0.41 | 0.0264 |
|  | Inbreds | 14 | 0.56 | 0.0262 |
| Flower development | Natural Selection | 4 | NA | NA |
|  | Domestication | 350 | 0.91 | 1.022-48 |
|  | Inbreds | 121 | 0.79 | 3.40e-20 |
| Response to auxin | Natural Selection | 3 | NA | NA |
|  | Domestication | 645 | 0.9 | 2.79e-91 |
|  | Inbreds | 239 | 0.78 | 2.30e-33 |
| Seed dormancy | Natural Selection | 3 | NA | NA |
|  | Domestication | 1067 | 0.78 | 4.36e-135 |
|  | Inbreds | 254 | 0.76 | 1.23e-37 |

**Table S3**

| **TFs group 1** | **TFs group 2** | **TFs group 1** | **TFs group 2** | **Interactions group 1** | **Interactions group 2** | **Effect size** | **Adjusted p value** |
| --- | --- | --- | --- | --- | --- | --- | --- |
| Additive | Dom-Rec | 65 | 110 | 2107 | 4555 | 0.58 | ~ 0 |
|  | P. Dom-Rec |  | 118 |  | 4513 | 0.51 | ~ 0 |
|  | Transgressive |  | 150 |  | 5051 | 0.39 | 4.44e-238 |
| Dom-Rec | P. Dom-Rec | 110 | 118 | 4555 | 4513 | 0.19 | 4.44e-77 |
|  | Transgressive |  | 150 |  | 5051 | 0.29 | 2.29e-179 |
| P. Dom-Rec | Transgressive | 118 | 150 | 4513 | 5051 | 0.12 | 1.61e-35 |

**Table S4**

| **Genetic cross** | **Tissue** | **Developmental stage** | **Growing conditions** | **Reference** |
| --- | --- | --- | --- | --- |
| **Switchgrass** | Leaf | Vegetative | Open experimental field | https://genome.cshlp.org/content/early/2016/03/07/gr.198135.115 |
| **Maize**  (W22-TIL3)  (Oh43-TIL3)  (B73-TIL3) | Ear | Reproductive | Growing chamber (12-hour dark-light) | https://journals.plos.org/plosgenetics/article?id=10.1371/journal.pgen.1004745 |
| **Chili pepper** | Fruit | Reproductive | Greenhouse | https://academic.oup.com/mbe/article/37/6/1593/5729999 |
| **Sunflower** | Leaf | Vegetative | Greenhouse | https://bmcgenomics.biomedcentral.com/articles/10.1186/1471-2164-14-342 |
| **Rice**  (indica-japonica)  (japonica-indica) | Leaf | Vegetative | Growing chamber (12-hour dark-light) | https://www.pnas.org/doi/abs/10.1073/pnas.1209297109 |
| **Arabidopsis**  (Col-C24)  (C24-Col)  (Col-Ler)  (Ler-Col) | Leaf | Vegetative | Growing chamber (12-hour dark-light) | https://www.pnas.org/doi/full/10.1073/pnas.1519926112 |
